# Supplementary material for: Identification of Freezing-Responsive microRNAs and Their Targets in Chinese Jujube by Small RNA and Degradome Sequencing
Source: Int J Mol Sci. 2026 Sep 4;27(17):7912. doi: 10.3390/ijms27177912 (PMC13566890; doi:10.3390/ijms27177912)
Supplement: Supplementary file 1 [file ijms-27-07912-s001.zip › Supplementary Material S2/novel_miR_227.pdf]

Provisional ID : novel_miR_227
Score total : 0.4
Score for star read(s) : -1.3
Score for read counts : 0
Score for mfe : 0.1
Score for randfold : 1.6
Score for cons. seed :
Total read count : 328
Mature read count : 328
Loop read count : 0
Star read count : 0

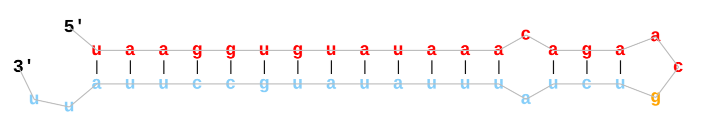

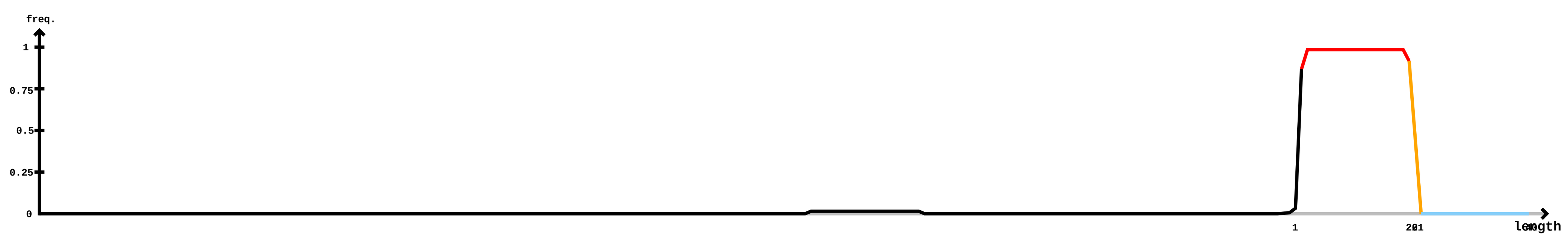

Mature Star

```
5'- cauaaaaaaagaaaaauuuaauuucucaagauauuugcuccgaaauuuguuauagcaucuauaacggccuuugauuuagguggacaaccgggcaaacaaacaucuacgagaauuaucuuaucgaccccccgaacaguacuauaagaauuaguacuugauuauauuugauuguuauucaauuucaaucagcagaaguaaauaaaaugaaauaagguguauaaacagaacgucuauuuauaugccuuauuuu -3'  exp
    ..............(((((.(((((.((((((.((.(((.((..((((((...((........(.((((......)))).)........)).))))))..))...))))).)))))).(((......)))..(((((((.......)))))))........((((((...........)))))).))))))))))).....((((((((((((((((.(((...))).))))))))))))))))  reads  mm  sample
    ...............................................................................................................................cccgaacaguacuauaaga........................................................................................................  5  0  LT-2
    ..............................................................................................................................................................................................................aaauaagguguauaaacagaac......................  1  0  LT-1
    ...............................................................................................................................................................................................................aauaagguguauaaacagaa.......................  1  0  LT-3
    ................................................................................................................................................................................................................auaagguguauaaacagaac......................  4  0  LT-2
    ................................................................................................................................................................................................................auaagguguauaaacagaac......................  2  0  LT-1
    ................................................................................................................................................................................................................auaagguguauaaacagaac......................  2  0  LT-3
    ................................................................................................................................................................................................................auaagguguauaaacagaac......................  1  0  JCK-3
    .................................................................................................................................................................................................................uaagguguauaaacagaa.......................  3  0  JT-2
    .................................................................................................................................................................................................................uaagguguauaaacagaa.......................  2  0  LT-2
    .................................................................................................................................................................................................................uaagguguauaaacagaa.......................  1  0  JT-3
    .................................................................................................................................................................................................................uaagguguauaaacagaC.......................  5  1  LCK-3
    .................................................................................................................................................................................................................uaagguguauaaacagaa.......................  2  0  JCK-2
    .................................................................................................................................................................................................................uaagguguauaaacagaa.......................  7  0  LT-3
    .................................................................................................................................................................................................................uNagguguauaaacagaa.......................  1  1  JT-2
    .................................................................................................................................................................................................................uaagguguauaaacagaa.......................  1  0  JCK-1
    .................................................................................................................................................................................................................Caagguguauaaacagaac......................  1  1  JCK-1
    .................................................................................................................................................................................................................uCagguguauaaacagaac......................  1  1  LT-1
    .................................................................................................................................................................................................................uaagguguauaaacagaac......................  26  0  JCK-3
    .................................................................................................................................................................................................................uaagguguauaaacagaac......................  14  0  JT-1
    .................................................................................................................................................................................................................uaagguguauaaacagaac......................  49  0  LCK-3
    .................................................................................................................................................................................................................uaagguguauaaacagaac......................  26  0  LT-2
    .................................................................................................................................................................................................................uaagguguauaaacagaac......................  62  0  LT-3
    .................................................................................................................................................................................................................uaagguguauaaacagaac......................  17  0  JCK-2
    .................................................................................................................................................................................................................uaagguguauaaacagaac......................  42  0  LT-1
    .................................................................................................................................................................................................................uaagguguauaaacagaac......................  17  0  JCK-1
    .................................................................................................................................................................................................................uaagguguauaaacagaacA.....................  2  1  LT-1
    ..................................................................................................................................................................................................................aagguguauaaacagaac......................  10  0  LCK-3
    ..................................................................................................................................................................................................................aagguguauaaacagaac......................  6  0  LT-3
    ..................................................................................................................................................................................................................aagguguauaaacagaac......................  9  0  LT-2
    ..................................................................................................................................................................................................................aagguguauaaacagaac......................  8  0  LT-1
    ..................................................................................................................................................................................................................aagguguauaaacagaac......................  2  0  JCK-3
    ..................................................................................................................................................................................................................aagguguauaaacagaacA.....................  1  1  JCK-3
    ..................................................................................................................................................................................................................aagguguauaaacagaacA.....................  1  1  JCK-1
    ..................................................................................................................................................................................................................aagguguauaaacagaacA.....................  2  1  JCK-2
```

Mature Star

cauaaaaaaagaaaaauuuaauuucucaagauauuugcuccgaaauuuguuauagcaucuauaacggccuuugauuuagguggacaaccgggcaaacaaacaucuacgagaauuaucuuaucgaccccccgaacaguacuauaagaauuaguacuugauuauauuugauuguuauucaauuucaaucagcagaaguaaauaaaaugaaauaagguguauaaacagaacgucuauuuauaugccuuauuuu
